# Pattern of genetic differentiation of an incipient speciation process: The case of the high Andean killifish *Orestias*

**DOI:** 10.1371/journal.pone.0170380

**Published:** 2017-02-28

**Authors:** Claudia Jimena Guerrero-Jiménez, Fabiola Peña, Pamela Morales, Marco Méndez, Michel Sallaberry, Irma Vila, Elie Poulin

**Affiliations:** 1 Departamento de Ciencias Ecológicas, Facultad de Ciencias, Universidad de Chile, Ñuñoa, Santiago, Región Metropolitana, Chile; 2 Escuela de Medicina Veterinaria, Facultad de Recursos Naturales y Medicina Veterinaria, Universidad Santo Tomás, Talca, Región del Maule Chile; 3 Escuela de Medicina Veterinaria, Facultad de Recursos Naturales y Ciencias Silvoagropecuarias, Universidad Iberoamericana de Ciencias y Tecnología, Santiago, Santiago, Región Metropolitana, Chile; 4 Faculty of Life Science, Utrecht University, Utrecht, Netherlands; 5 Instituto de Ecología y Biodiversidad de Chile, Universidad de Chile, Ñuñoa, Santiago, Región Metropolitana, Chile; University of Padova, ITALY

## Abstract

During the Pleistocene and Holocene, the southwest Andean Altiplano (17°-22°S) was affected by repeated fluctuations in water levels, high volcanic activity and major tectonic movements. In the early Holocene the humid Tauca phase shifted to the arid conditions that have lasted until the present, producing endorheic rivers, lakes, lagoons and wetlands. The endemic fish *Orestias* (Cyprinodontidae) represents a good model to observe the genetic differentiation that characterizes an incipient speciation process in allopatry since the morphospecies described inhabit a restricted geographic area, with present habitat fragmentation. The genetic diversity and population structure of four endemic morphospecies of *Orestias* (Cyprinodontidae) found in the Lauca National Park (LNP) analyzed with mitochondrial markers (Control Region) and eight microsatellites, revealed the existence of genetic groups that matches the fragmentation of these systems. High values of genetic and phylogeographic differentiation indices were observed between Chungará Lake and Piacota lagoon. The group composed of the Lauca River, Copapujo and Chuviri wetlands sampling sites showed a clear signal of expansion, with a star-like haplotype network. Levels of genetic differentiation were lower than in Chungará and Piacota, suggesting that these localities would have differentiated after the bottlenecks linked to the collapse of Parinacota volcano. The Parinacota sample showed a population signal that differed from the other localities revealing greater genetic diversity and a disperse network, presenting haplotypes shared with other LNP localities. A mixing pattern of the different genetic groups was evident using the microsatellite markers. The chronology of the vicariance events in LNP may indicate that the partition process of the *Orestias* populations was gradual. Considering this, and in view of the genetic results, we may conclude that the morphospecies from LNP are populations in ongoing differentiation process.

## Introduction

Speciation is a continuous process during which differentiation and reproductive isolation are established [1]. In allopatry, geographic isolation impedes gene flow between local populations, while microevolutionary processes such as local adaptation and genetic drift proceed [2,3,4]. These processes enhance morphological variations [5,6], high genetic structuring of populations [7,8,9] and differences in karyotypes as a consequence of the new chromosome rearrangements that may cause reproductive incompatibility, [10,11], among others [12,13].

Fishes exhibit great morphological and ecological diversity, inhabiting very diverse aquatic habitats. These characteristics have made them a speciose and diversified group. They are used for the study of processes implicit in the generation of species [14,15,16,17,18,19]. Some fish show unusually high speciation rates and have received special attention. The family Cichlidae is the classical model to study an adaptive radiation and diversification in lake systems [20,21]. One example of this group is haplochromine cichlids from the Lake Victoria region in Africa, where there are species with low genetic differentiation but with different morphological characteristics. It is postulated that the diversification would be related to the variation in the water level of the large lakes produced 18000–15000 YBP (years before present); cichlids that had been isolated in small nearby lakes and creeks would then have colonized the large lakes [20, 22, 23]. Barluenga & Meyer [7] studied the speciation processes of the Midas cichlid in the lakes of Nicaragua; they found that the large lakes had genetically more ancestral populations, while species ensembles that inhabit crater lakes were younger and had less genetic variation. In spite of their recent origin (<25000 YBP), the isolation of the populations that inhabit crater lakes combined with the selective pressures to which they are exposed (physicochemical conditions of the water, size and depth) suggest a speciation process in these lakes.

Similar to the cichlids in Africa and Central America, *Orestias* is a speciose group of biogeographic interest due to its endemism and adaptation to the aquatic systems of the Altiplano [24,25,26]. This extensive flatland between 9° S and 22° S at more than 3000 m elevation has diverse aquatic systems formed since the Quaternary [27,28,29,30]. The current distribution of *Orestias* extends from Lago Lascha (9°S) in the center of Perú to the Salar de Ascotán in northern Chile (22°S) [26]. Of the 44 species described, 23 inhabit Titicaca lake (Bolivia-Peru), while the other 21 species inhabit rivers, lagoons, lakes, wetlands and salt pans of the Altiplano.

The long-term climate variations in this region, which have included alternation of wet and dry periods, generated a succession of paleolakes that covered extensive areas and included several vast salt flats and localized and fragmented watersheds [31]. Simultaneously, intense volcanic activity and strong tectonic movements formed large closed watersheds in the eastern Altiplano (currently Titicaca lake, Desaguadero river and the Uyuni and Coipasa salt pans), and in the western part, watersheds with small, isolated lakes, wetlands, salt pans and rivers [32, 33]. These geological and climatic changes, along with changes in the size of lakes, determined the current distribution of *Orestias* [26]; in this scenario the populations of fish that inhabit the lakes were divided into multiple isolated populations, producing different degrees of divergence and finally different species [34].

**Fig 1:**
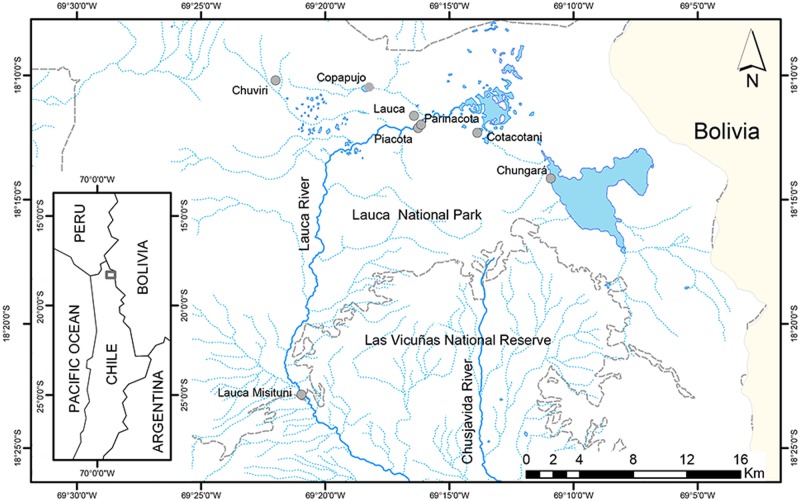
Map of localities sampled at Lauca National Park Chile.

The highest region of the southwest Altiplano is found in the Lauca National Park, Chile (LNP), which is mostly above 4000 m elevation; the paleolakes did not cover this region. However, advances and retreats of glaciers and volcanic events produced changes in the landscape and fragmentation of the watersheds [35,36,37,38]. During the Holocene, about 12500 YBP, the collapse of the cone of the Parinacota volcano modified the watershed of the Lauca River, in different aquatic systems such as lakes, lagoons and wetlands [35,38]. Paleoclimate records indicate an increase in volcanic activity from 7800 to 1000 YBP, along with the establishment of a dry period that maintained the watersheds of Lauca and Chungará isolated until now [36,38]. This fragmentation would have produced the isolation of various populations, allowing differentiation. The four morphospecies described reflect this process: *O*. *chungarensis* Vila & Pinto 1986 in Chungará lake, *O*. *piacotensis* Vila 2006 in the Piacota lagoon, *O*. *parinacotensis* Arratia 1982 in the Parinacota wetland and *O*. *laucaensis* Arratia 1982 in the Lauca River and the Cotacotani Lagoon (Fig 1).

Studies of the species of *Orestias* have been mainly focused on morphological analysis in the context of traditional systematics, initially associated with taxonomic descriptions [39, 40, 41]. More recently, Vila et al 2010 [26] compared the morphology of *Orestias* that inhabit the Chilean Altiplano using linear and meristic (rays in fin, number of vertebrae) measurements; their results showed that some nominal species in the LNP were differentiated morphologically, (*O*. *laucaensis* and *O*. *chungarensis*), although meristic data showed high overlap between nominal species and could not distinguish them. Recent trophic studies of *Orestias* in the LNP watersheds showed low prey richness and dietary range, which was related to the reduced size and shallow depth of these systems, with mostly microinvertebrates inhabiting the macrophyte belt. Thus, the local populations of *Orestias* showed similar dietary habits associated with prey availability [42, 43].

From a phylogenetic point of view, a previous study suggested that diversification of *Orestias* in the southern Altiplano may be linked to historical vicariant events and contemporary variation in water level [34]. In this study the four morphospecies of the LNP formed a monophyletic group with good statistical support, however, they do not form monophyletic clades within this lineage, challenging the validity of these taxa under a phylogenic species concept [34]. A preliminary review of the number and morphology of chromosomes and the distribution of constitutive heterochromatin in the karyotypes of the morphospecies of the LNP compared to the other Chilean species showed that these four taxa form a distinct chromosome lineage, characterized by abundant constitutive heterochromatin in chromosome pair one. However, in contrast with the phylogenetic evidence, the four LNP morphospecies exhibited karyotype differences in the number of telocentric and subtelocentric chromosomes and microchromosomes [26]. In spite of the karyotype differences found, studies have not been performed to test the existence of reproductive barriers among these taxa.

In summary, the different analyses of the nominal species of the LNP have shown contrasting results in terms of their differentiation. Morphometric measurements and karyotypes have allowed distinguishing them as separate entities, while meristic measurements and molecular phylogeny have indicated that these nominal species do not constitute distinguishable groups. This discrepancy between different morphological and genetic approximations has been interpreted to be the result of incomplete sorting [34, 44], that is not enough time has elapsed for allopatric genetic differentiation to be translated into reciprocal monophyly between these taxa. In this context, our study adds more samples and uses more variable markers (Dloop, microsatellites against Cyt b, ND2 in the previous study) to further investigate the genetic relationship among LNP morphospecies.

In the present study, we hypothesize that all four morphospecies would have been formerly part of a unique Lauca River basin population that was fragmented in different bodies of water due to an intense volcanic activity during Holocene time. The presence of four *Orestias* morphospecies described in a restricted geographic area, each one occupying a different body of water, would therefore reflect an incipient allopatric speciation process. Under this hypothesis, we would expect to find genetic differentiation between the different bodies of water, at least for the most isolated ones, and an incomplete lineage sorting due to the short time elapsed since the fragmentation process.

## Materials and method

### Sampling sites and DNA isolation

The samples (n = 270) were collected in eight localities of the Lauca National Park (LNP) including the sites where the four morphospecies of *Orestias* were described. These locations represent the diverse aquatic systems shaped by the fragmentation of the Lauca River (Fig 1, Table 1, S1 File). Chungará Lake and Piacota lagoon represent the most isolated sites while do not connect with any other body of water. In contrast, the aquatic systems such as the wetlands of Parinacota, Chuviri, Copapujo as well as Cotacotani lagoon may be temporarily connected with Lauca River [45]. All specimens were euthanized using 100 mg/L tricaine methanosulfonate and fixed in 95% ethanol. DNA was extracted using the saline method [46]. For this study, samples were collected under permit from the Subsecretaria de Pesca, Chile, Resolución Exenta # 2231, 19 August 2011, and approved by the Bioethical Committee at the Universidad de Chile.

**Table 1:** Sampling localities at Lauca National Park. N: sample size. Habitats: Type of aquatic systems.

Localities	N	Geographical coordinates	Habitats	Morphospecies
Piacota	46	S18.19984 W69.26905	Lagoon	*O*. *piacota*
Parinacota	29	S18.20200 W69.27105	Wetland	*O*. *parinacotensis*
Chungará	32	S18.23820 W69.18296	Lake	*O*.*chungarensis*
Cotacotani	44	S18.20530 W69.23126	Lagoon	*O*. *laucaensis*
Lauca	37	S18.19379 W69.27360	River	*O*. *laucaensis*
Misituni	48	S18.38074 W69.34920	River	*O*. *laucaensis*
Chuviri	23	S 18,16925 W 69,33481	Wetland	*Orestias sp*
Copapujo	11	S 18,16925 W 69,30785	Wetland	*Orestias sp*

### Mitochondrial DNA (D-loop)

The control region of mitochondrial DNA (D-loop) was amplified by PCR using specific primers for the genus: (Forward 5’ ACC CCT AAC TCC CAA AGC T 3’) (Reverse 5’ TGA TAG TAA AGT CAG GAC CAA 3’) [47]. The PCR reaction was standardized in a total volume of 25 μl 10 X PCR buffer (50mM KCl, 10mM Tris-HCl, pH 8.0), MgCl_2_ 50 mM, each primer 10 pm/μl, dNTP 2.5 mM, 0.5 μl Taq (Invitrogen), 9 μl ultrapure water, 1 μl DMSO and 10 ng/μl DNA.

The PCR cycle consisted of an initial denaturation for 5 min at 94°C, followed by 38 cycles of 45 sec at 94°C, 90 sec at 66°C and 90 sec at 72°C, with a final extension of 10 min at 72°C. PCR products were visualized in 2% agarose gel stained with ethidium bromide. Amplification products were sent for sequencing to Macrogen Inc. (South Korea). D-loop sequences were aligned, edited and assembled using the Proseq v.2.9.1 program [48]. Using the D-loop sequences we estimated diversity indices for each locality, including number of haplotypes (K), number of polymorphic sites (S), haplotype diversity (H), mean number of differences between pairs of sequences (Π) and nucleotide diversity (π) in the DnaSP v.5 program [49]. Coalescent analysis was be based on single locus, the genealogical relations among haplotypes were graphed by constructing an haplotype network using the median-joining algorithm implemented in the Network version 4.501 software [50].

The genetic structure as a function of the geographic component was analyzed using SAMOVA (Spatial Analysis of Molecular VAriance), which identifies the groups of genetically homogeneous and geographically close localities that maximize the variance of genetic diversity among these groups [51]. To estimate the level of genetic differentiation between the different groups identified by SAMOVA, we used two indices: F_ST_, based on the differences in haplotype frequencies and Φ_ST_, based on the number of differences between pairs of sequences, with the Arlequin v.3.5.1.3 program [52]. The statistical significance of F_ST_ and Φ_ST_ was based on 10000 permutations; we used the false discovery rate [53] correction for multiple tests. We used the PERMUT program [54] to calculate the estimators of population differentiation G_ST_ and N_ST_ among groups. N_ST_ takes into account the genetic distance between haplotypes, while G_ST_ only considers the difference in frequency. N_ST_ significantly greater than G_ST_ suggests phylogeographic structure. We performed 1000 permutations to determine its significance; if more than 5% of the permuted values of N_ST_ are greater than G_ST_ the null hypothesis G_ST_ = N_ST_ is rejected.

We performed the Tajima [55] and Fu [56] tests in each genetic group defined by SAMOVA, to detect deviations from the mutation-drift equilibrium expected with the Wright-Fisher model that could indicate population expansions or bottlenecks under the assumption that the sequence variants are selectively neutral.

We used three different models to evaluate past demographic changes. First, we constructed the mismatch distribution for each group, and when pertinent we estimated the demographic parameters of the expansion using the Sudden demographic expansion model of Schneider & Excoffier [57] with the Arlequin v.3.5 program. In the cases where this match did occur, we estimated the parameter of demographic expansion Tau: *τ* = 2*tμ*, which is the time when growth began measured in mutational time. t is the time in generations and μ is the mutation rate per sequence per generation. The initial value of Theta is given by *θ*_0_ = 2*N*_*i*_*μ* where N_e_0 is the initial effective population size estimated before population growth, while the final value is assumed to be infinite, θ_1_ = ∞, according with the Sudden demographic expansion model of Schneider & Excoffier [57].

Second, we reconstructed the demographic history of each genetic group using the LAMARC 2.1.5 program [58]. This program constructs coalescence trees using maximum likelihood; from these trees several population parameters are estimated. The growth rate g and the value of θ are estimated with the assumption of exponential growth, following *θ*_*t*_ = *θ*_0_*e*^−*gt*^, where θ_t_ is the value of θ at time t and θ_0_ is the current value of θ. Using these parameter s we estimated the time since the beginning of demographic expansion in units of mutational time. The time of the initiation of expansion was estimated as the moment when θ_t_ was 1% of θ_0_, as recommended by the author of the program [58].

Third, the demographic history of the groups was inferred using a Bayesian Skyline Plot (BSP) analysis with the BEAST program version 1.7.1 [59], which assume a demographic model just not one of constant size. The assumption used to generate coalescence trees was a relaxed molecular clock; trees were generated with the exponential distribution. These analyses were performed with MCMC chains of 300 million iterations; parameters and trees were sampled every 1000 generations, discarding the first 100 million generations as burn-in. The results were graphed with the Tracer program version 1.7 [60].

The only study for the estimation of freshwater fish mutations rates including time dependency in molecular rates was introduced by Ho (2005). He dates changes in river drainages and isolation of fish populations to document rates of mitochondrial DNA change over a range of temporal scales. He utilizes precise spatio-temporal disruptions of linear freshwater systems and hence avoids many of the limitations associated with typical DNA calibration methods involving fossil data.

We used a mutation rate of 6% per million years proposed by Burridge et al. [61]; their study were based on a robust calibration system applied to closely related taxa in the Galaxidae.

### Microsatellites

We amplified eight microsatellite loci, originally optimized in *O*. *agassii* of Bolivia by Esquer-Garrigos et al. [62], for each of the localities (Table A in S2 File). PCR reactions were standardized in a 10 μl volume that contained: 5X PCR buffer (50mM KCl, 10mM Tris-HCl, pH 8.0), MgCl_2_ 25 mM, BSA 0,1mg/μl, primers 50 pm/μl, dNTP 2.5 mM, 0.1 μl Taq (Promega Inc.) and 50 ng/μl DNA. The PCR conditions were: initial denaturation for 5 min at 94°C, followed by 35 cycles of 30 sec at 92°C, 30 sec at the locus-specific aligning temperature (T° a) (Table A in S2 File) and extension at 72°C for 60 sec, with a final extension of 20 min at 72°C. PCR products were visualized in 3% agarose gels stained with ethidium bromide to verify positive amplification. Amplification products were sent for genotyping to Macrogen Inc. (South Korea). The analysis of fragments was performed in the software GENEMAPPER 3.0 (Applied Biosystems). For each locus we calculated allele frequencies, observed (Ho) and expected (He) heterozygosity with the Genetix v. 4.05 software [63]. The values of F_IS_ were calculated in Genetix; we performed an exact test in Genepop 4.2 to determine possible deviations from Hardy-Weinberg equilibrium for each locus and locality [63, 64].

The degree of genetic structure between localities was analyzed by calculating F_ST_ [65] between pairs of localities with 10000 permutations in Genetix, using the false discovery rate for multiple tests. Localities that didn't show any signal of genetic difference (Fst: NS) were assigned to a same population. To determine if the genotyped individuals correspond to the population groups defined in the structure analysis, we performed an assignment test with the Geneclass 2.0 program [66], using the frequency method described by Paetkau et al. [67].

We then performed a Bayesian analysis to infer the number of genetic populations (K) with individual genotype data using the software STRUCTURE V.2.0 [68]. The program was run 10 times, discarding the first 10000 iterations as burn-in, followed by 500,000 MCMC iterations; these parameters were calculated for each value of K under the admixture model and correlated allele frequencies between populations. The value of α, the degree of mixture between the K populations, was evaluated for each population separately. We analyzed K values from 1 to 8 population based on LnP (D), the logarithm of the likelihood of the observed data as a function of K. We also calculated ΔK based on the rate of the change of log likelihood between successive values of K, to identify the number of genetic cluster in the data set [69].

## Results

### Mitochondrial control region

**Fig 2:**
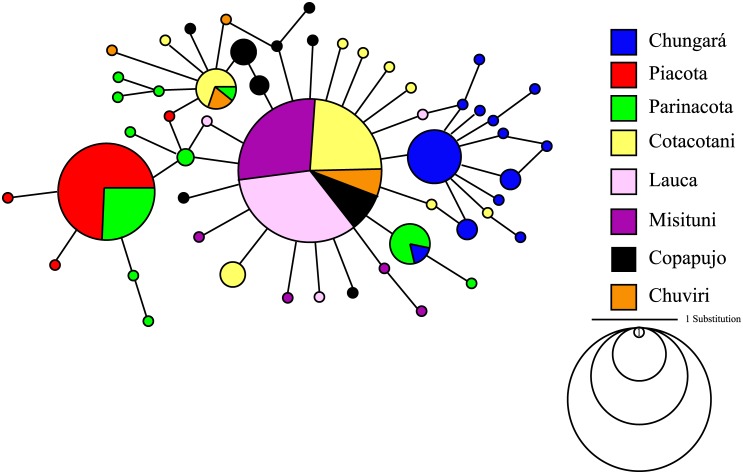
Median joining network of mitochondrial control region haplotypes including 270 mtDNA d-loop control region sequences in the 8 sampling sites. Each haplotype is represented by a colored circle indicating the main site where it was collected; the size of the circle is proportional to its frequency in the whole sampling effort.

We obtained a total of 270 samples in the eight sampling sites representing 890 nucleotide positions (GenBank Accesion Numbers BankIt1931003:KX498091-KX498358). The descriptive indices of genetic diversity showed 42 polymorphic sites (S); the number of haplotypes was K = 51; the haplotype diversity was Hd = 0.747 and the mean number of differences between pairs of sequences was Π = 1.593 (Table 2). The sample of Piacota lagoon had the smallest number of haplotypes (K = 4 with N = 46), the lowest haplotype diversity (Hd = 0.128) and the least number of differences between pairs of sequences (Π = 0.174). By contrast, the sample of the Parinacota wetland showed a high number of haplotypes (K = 11 with N = 29), the greatest haplotype diversity (Hd = 0.79) and the largest number of differences between pairs of sequences (Π = 2.192) (Table 2). The haplotype network revealed a small but clear differentiation between Piacota and Chungará (4 substitutions); both sites had a central haplotype with short branches. By contrast, Parinacota showed haplotypes much more dispersed in the network (Fig 2).

**Table 2:** Genetic diversity indices for the D-loop in different samples of *Orestias*. N: sample size; S: Polymorphic sites; K: Number of haplotypes; []: Rarefaction of the number of haplotypes; Hd: Haplotype diversity; π: Nucleotide diversity; Π: Mean number of differences between pairs of sequences.

Samples	N	S	K	Hd	π	Π
Chungará *O*.*chungarensis*	32	13	13 [5.590 ± 1.294]	0.688	0.0013	1.159
Parinacota *O*.*parinacotensis*	29	10	11 [5.976 ± 1.129]	0.793	0.00246	2.192
Piacota *O*.*piacotensis*	46	4	4 [1.717 ± 0.727]	0.128	0.0002	0.174
Cotacotani *O*.*laucaensis*	44	9	9 [3.821± 1.100]	0.518	0.00075	0.668
Lauca *O*.*laucaensis*	37	3	4 [1.89 ± 0.76]	0.158	0.00024	0.213
Misituni *O*.*laucaensis*	48	6	7 [2.467± 0.924]	0.340	0.00041	0.368
Copapujo *O*.*sp*	11	4	4	0.600	0.00118	1.055
Chuviri *O*.*sp*	23	11	9 [5.552±1.0869]	0.755	0.00182	1.621
Total	270	42	51	0.747	0.0018	1.593

The SAMOVA showed that the variation between groups was maximized with four groups (59.92%); the corresponding variance between sample within groups was low (2.14%) but significant. The four genetic groups in the LNP were: Group 1: Chungará; Group 2: Parinacota; Group 3: Piacota and Group 4: Lauca, Misituni, Cotacotani, Copapujo and Chuviri (Table B in S2 File). The values of Φ_ST_ and F_ST_ among groups were low but significant for the majority of the comparisons (Table 3a and 3b).

**Table 3:** Genetic differentiation among groups defined by SAMOVA based on (a) differences of haplotype frequencies F_ST_ and (b) number of differences among sequencesΦ_ST_. Group1: Chungará; Group 2: Parinacota; Group 3: Piacota; Group 4: Lauca, Misituni, Cotacotani, Chuviri and Copapujo. Corrected significance is shown above the diagonal *** P<0.0001.

A				
F_ST_	**GROUP 1**	**GROUP 2**	**GROUP 3**	**GROUP 4**
**GROUP 1**		0.000***	0.000***	0.000***
**GROUP 2**	0.48		0.000***	0.000***
**GROUP 3**	0.81	0.32		0.000***
**GROUP 4**	0.58	0.45	0.79	
B				
Φ_ST_	**GROUP 1**	**GROUP 2**	**GROUP 3**	**GROUP 4**
**GROUP 1**		0.000***	0.000***	0.000***
**GROUP 2**	0.25		0.000***	0.000***
**GROUP 3**	0.62	0.33		0.000***
**GROUP 4**	0.49	0.54	0.67	

The structure index Nst (0.576) based on the number of differences between pairs of sequences was significantly greater than Gst (0.462) calculated from the haplotype frequencies (p = 0.0034), indicating the existence of a phylogeographic structure among the groups of the LNP.

**Fig 3:**
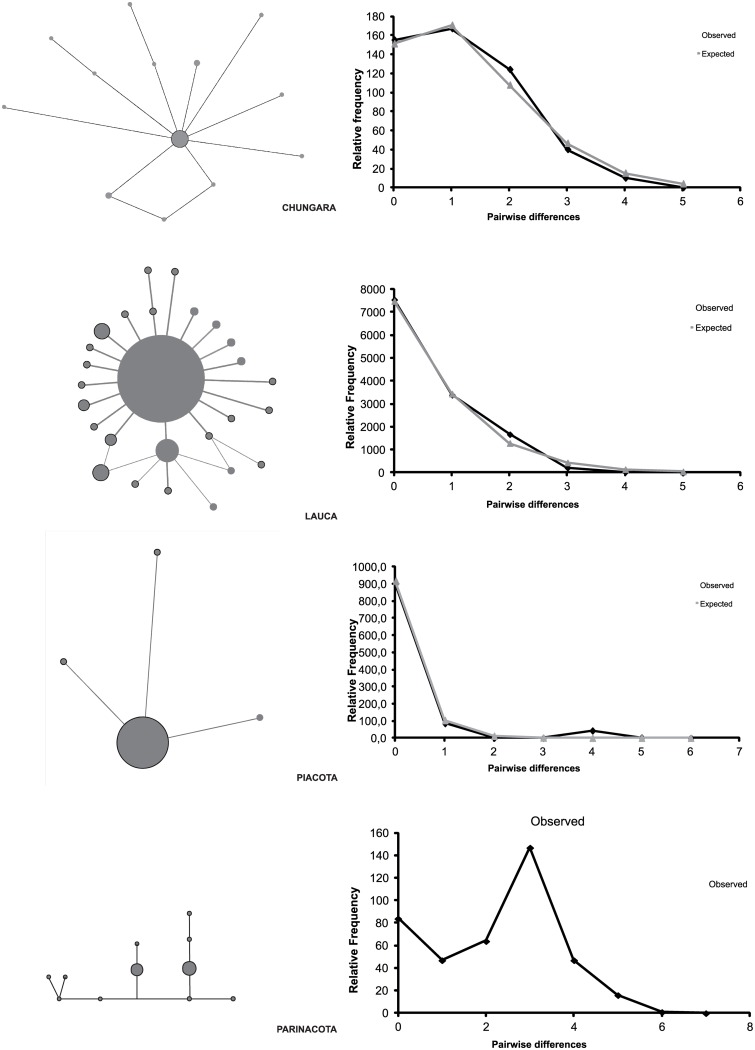
Median joining haplotype networks and the distribution of pairwise differences between haplotypes (mismatch distribution) analyses for each group obtained by SAMOVA. a) Chungará b) Lauca Group c) Piacota d) Parinacota.

Tajima’s D index was negative but not significant for Parinacota. In concordance, the mismatch distribution for the Parinacota group was multimodal (Fig 3) and didn’t show any evidence of demographic expansion, i.e, no deviation to mutation-drift equilibrium. Demographic inference analyses were performed only on groups which showed negative and significant value of Tajima’s D index as well as unimodal mismatch distribution: Chungará, Piacota and the Lauca groups. The parameters estimated for Chungará under the Sudden demographic expansion model (τ = 1.67[0–2.24]) indicate that the expansion would have occurred 12000 YBP, with values of Ν_e_0 = 0 that indicate the reduced initial effective population size in the locality (Table 4). The parameters of the model for Piacota and the Lauca group showed that expansion beginning 3200 YBP for the Lauca group and 1600 YBP for Piacota (Table 4, Fig 3).

**Table 4:** Estimation of effective population sizes and expansion times under instantaneous, sudden demographic expansion and exponential growth models of the different genetic groups with unimodal distribution of *Orestias* in the Lauca National Park. Ne_i_: Initial effective population size; Ne_f_: Final effective population size.[]: confidence intervals.

	Sudden demographic expansion model Schneider & Excoffier (1999). Arlequin v.3.5			
Groups	τ = 2μt	N_e_0	N_e_1	Time (years)
Chungará	1.67[0–2.24]	0	90458.8015	12378[0–21012]
Piacota	0,174[0–0,8]	0	1189.138577	1629[0–7490]
Lauca-group	0.342[0–2.02]	4606.741573	9859.550562	3202[0–19000]
	Model of coalescence by likelihood Kuhner (2006) LAMARC v.2.1.8			
Groups	G	Ne_i_	Ne_f_	Time
Chungará	10139.72[5208.93–18516.47]	29977[3999–2685565]	3716067[391475–228570333]	7800[4000–14500]
Piacota	17585.06[12234.67–22092.12]	615[4928–408916]	59517[37816–408916]	4350[3000–6000]
Lauca-group	14701.86[19292.9–11128.56]	23244[7996–51267]	2216350[76637–4334717]	5200[3500–6500]

Assuming exponential growth, the rates of growth G and population sizes obtained using the maximum likelihood analysis (LAMARC 2.1.5 program) indicated that the growth of the populations of Chungará (Ne_i_ = 30000; Ne_f_ = 370000), the Lauca group (Ne_i_ = 24000; Ne_f_ = 220000) began from 8000–4300 YBP with effective sizes of one or two orders of magnitude lower in Piacota (Ne_i_ = 700; Ne_f_ = 60000) (Table 4).

**Fig 4:**
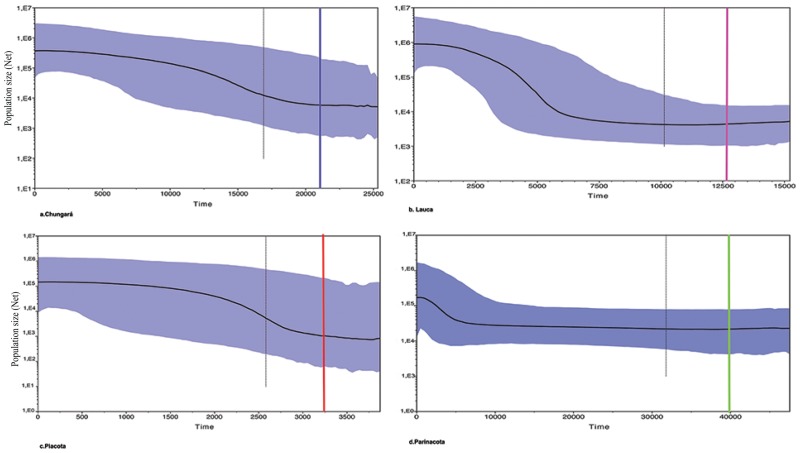
Historical demographic trends of the effective population size (Ne) constructed using a Bayesian skyline plot approach based on control region haplotypes of groups of *Orestias* (a. Chungará, b. Lauca, c. Piacota, d. Parinacota). The y-axis is the product of effective population size (Ne) and generation length in a log scale (Population size (N_e_t)) while the x-axis is the time before present (years). The median estimate (black solid line) and 95% highest probability density (HPD) limits are shown (in purple). The color lines represent the time of the most recent ancestor (TRCMA) in the *Orestias* population. The thin dashed line represents time for the expansion in the species.

The Bayesian skyline plots for the genetic groups also showed patterns of population growth and differences in expansion times. The expansion began approximately 5000 YBP in Parinacota, with an increase of population size of 40000 and a mean of 100000 individuals (Fig 4d); the expansion in Chungará began 17000 YBP and the Lauca group 8000 YBP, with an increase in effective population size from 100000 to 1 million individuals respectively (Fig 4a and 4b). In Piacota the increase in mean population size was much less than in the other localities (from 5000 to 100000 individuals), with that expansion beginning 2500 YBP (Fig 4c). The mean time to the most recent common ancestor (TMRCA) of the groups is shown in Table C in S2 File.

### Microsatellites

We obtained multilocus genotypes in 261 of 270 samples of *Orestias* the samples for 8 loci (Table E in S2 File). Loci A116, D110 and A106 had the largest numbers (14 to 15) of alleles. Locus C102 had the smallest number (2 or 3) of alleles in all localities. Global evaluation of the microsatellite data set using Micro-checker indicated no evidence of non-amplified (null) alleles. Further tests for errors in the data showed no evidence of stuttering or large allele dropout. No significant deviations from Hardy—Weinberg equilibrium across populations were detected. No linkage disequilibrium was found between loci per population after Bonferroni correction.

All F_ST_ between pairs of samples were significant with and without the FDR correction, except for Lauca/Misituni and Cotacotani/Chuviri/Copapujo (Table 5). The sampling site that did not have significant differences was grouped in the Lauca (Lauca-Misituni) and the CCC (Cotacotani-Chuviri-Copapujo) populations.

**Table 5:** Measures of genetic differentiation (F_ST_) between pairs of *Orestias* populations based on information of 8 microsatellite loci. Above diagonal; Bonferroni-corrected degrees of significance. *** = P<0.0001. NS = Not significant.

FST	Chungará	Chuviri	Copapujo	Cotacotani	Lauca	Misituni	Parinacota	Piacota
Chungará		***	***	***	***	***	***	***
Chuviri	0.15434		NS	NS	***	***	***	***
Copapujo	0.15285	0.00826		NS	***	***	***	***
Cotacotani	0.16906	0.0326	0.02221		***	***	***	***
Lauca	0.09261	0.0975	0.11751	0.12753		NS	***	***
Misituni	0.10249	0.078	0.08711	0.08042	0.00911		***	***
Parinacota	0.11766	0.07297	0.10694	0.08887	0.08734	0.07164		***
Piacota	0.22205	0.21408	0.25058	0.20922	0.13100	0.12813	0.09148	

The descriptive indices of genetic diversity of the 8 microsatellite loci are shown in Table 6. The mean number of alleles per locus was similar in all populations; the largest number was in the CCC population with 9.3 alleles and the smallest number was in Piacota with 5 alleles. Parinacota had the highest allelic richness (6.6) and Piacota the smallest richness (4.1). F_IS_ values were not significantly different from zero (*p* values > 0.05) for any locus in any population (Table D in S2 File). Pairwise comparisons among loci did not show any linkage disequilibrium for population-specific analyses (*p* values > 0.05). Intermediate values of heterozygosity (0.47–0.60) were found for all populations; Chungará had the greatest mean heterozygosity (0.60) and Piacota had the lowest (0.47).

**Table 6:** Genetic diversity measures for 8 microsatellite loci for genetic populations of *Orestias* of the LNP. N = Number of individuals analyzed per locus; A: Mean number of alleles per locus; H_obs_: Observed heterozygosity; H_exp_: Expected heterozygosity; F_IS_: Endogamy index; Ne_C_: Contemporary effective population size; AR: Allelic richness. Chung: Chungará; CCC: Chuviri.-Copapujo- Cotacotani; Lau: Lauca group; Pari: Parinacota; Pia: Piacota. Braquets []: Standard deviation. NS = not significant.

Populations	N	A	AR	H_obs_	H_exp_	F_IS_	Ne_C_
CHUNG	24	6.5[3.7]	6.30[3.5]	0.63	0.60	-0.04^NS^	611
CCC	78	9.3[4.1]	6.43[4.0]	0.47	0.51	0.08^NS^	769
LAU	76	7.4[4.1]	5.61[3.8]	0.50	0.53	0.06^NS^	166.3
PARI	31	7.4 [(4.6]	6.60[3.7]	0.52	0.57	0.10^NS^	47.2
PIA	48	5.0[2.1]	4.10[1.8]	0.46	0.47	0.04^NS^	17.7

Individual assignment (GeneClass) to the populations delimited by pairwise Fst analysis showed that the greatest percentage of correct assignments was found in the Chungará and Piacota populations, with 100% and 96%, respectively. In the CCC and Lauca populations the correct assignments were 75% and 76%, respectively; the incorrectly assigned individuals of these two populations were assigned to the other population. Parinacota had the lowest percentage of correct assignments (68%); individuals incorrectly assigned were mainly assigned to CCC (12%) and Lauca group (12%).

**Fig 5:**
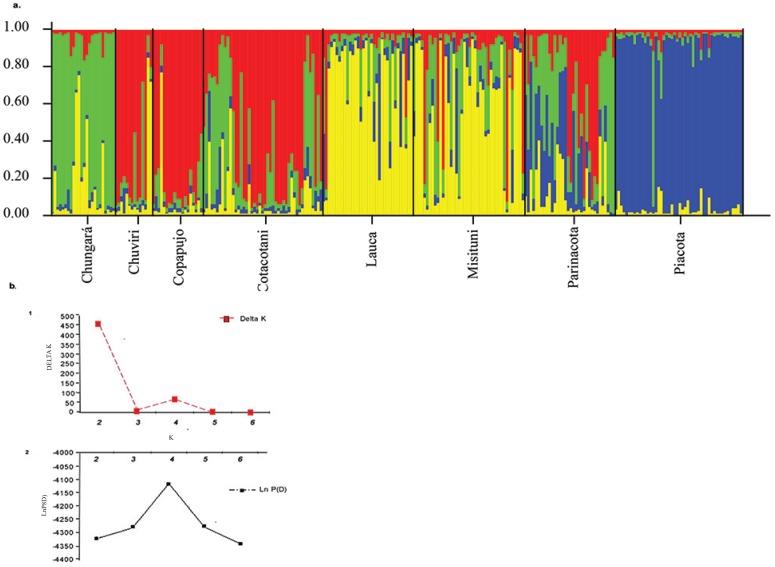
a. Results of the admixture analysis of *Orestias* for K = 4. Each individual is represented by a thin vertical line, which is partitioned into K colored segments that represent the individual’s estimated membership probabilities in K clusters. Black lines separate individuals of different populations. Populations are labeled below the plot. b. ΔK (1) and (2) ln (P) analysis.

The STRUCTURE analysis found the lowest value of LnP for K = 4 (Fig 5b), thus indicating four genetic clusters. The first group was composed mostly of individuals from Lago Chungará; the second group with individuals from the Cotacotani lagoons and the wetlands of Chuviri and Copapujo; the third group with individuals from the two river sites (Lauca and Misituni) and the fourth of individuals from the Piacota lagoon (Fig 5a). Although each locality had a predominance of only one genetic group (especially Chungará and Piacota), the individuals collected in the Parinacota wetland belonged indiscriminately to the different groups and showed high levels of admixture (Fig 5a).

## Discussion

Our results showed the existence of various genetic groups in the localities of the LNP that coincide with the fragmentation of the aquatic systems due to the volcanic and climate events that originated closed systems such as Lago Chungará and the Piacota lagoon [37, 38]. Genetic analyses performed with mtDNA and microsatellites coincided in separating clearly Chungará and Piacota, which are the most disconnected systems in the study area (Fig 1). This pattern is supported by the high values of genetic and phylogeographic differentiation between these two systems and with the rest of the localities (Tables 2, 3 and 6). These two localities harbor different morphospecies, *O*. *chungarensis* in Lake Chungará and *O*. *piacotensis* in Piacota lagoon.

The populations of these two localities show signs of growth after 12000 YBP, which may be related to the main volcanic events that occurred in the Pleistocene-Holocene boundary, which originated these lentic systems. The formation of the Chungará subwatershed (12000 YBP) as a product of the collapse of the Parinacota volcano isolated Lago Chungará from the Lauca watershed, which has probably lasted until the present. The low genetic variation of Chungará population, in particular the low number of differences between haplotypes, may indicate a founder effect during the separation and later appearance of new haplotypes during population growth in this large lake. The closed system of the Piacota lagoon has been disconnected from the Parinacota wetland during the arid period of the mid-Holocene (8000–4000 YBP). The small size of the lagoon would have limited the population size of *Orestias* (S3 Table), thus genetic drift may have acted more strongly, retaining a smaller number of new haplotypes. This difference in diversity between lakes of different sizes has also been documented in cichlids in the region of Lake Victoria in Africa [19, 23] and in the lakes of Nicaragua [7]. In these cases, the genetic diversity be associated with the size and depth of the lakes; populations in larger, older lakes have greater diversity than smaller, more recently formed lakes [7, 19].

The analysis of mitochondrial sequences identified a third group including the two sites of the Lauca river with those of Cotacotani lagoon and the wetlands of Copapujo and Chuviri. In the same way as the previous groups, this group shows a clear sign of expansion illustrated by a star-like haplotype network. During the volcanic processes (12000–4000 YBP) the Lauca river was probably affected by landslides from the collapse of the volcano dome, the pyroclastic rain, lahars and inundations, which may have generated population bottlenecks as has been reported for recent eruptions in other parts of Chile [70]. The analyses of genetic differentiation and assignment with microsatellite markers separated this group into two, one corresponding to the sites from the Lauca river and the other to the wetland of Copapujo and Chuviri and Cotacotani lagoons. However, the levels of genetic differentiation of the later two groups (Lauca River and CCC) appear to be lower than those estimated for the populations of Chungará and Piacota, suggesting that these localities would have differentiated after the bottlenecks linked to the collapse of Parinacota volcano.

The population of Parinacota showed a different population signal than the rest of the localities. Based on mitochondrial sequences showed greater genetic diversity and a disperse network with the presence of low-frequency haplotypes (Fig 3), several of them were shared with other localities of the LNP (Fig 2). The star-like haplotypes observed in the other localities may thus reflect founder effects associated with bottlenecks or recolonization events from the Parinacota population (Fig 3). In this scenario, the Parinacota site, that was an integral part of the Lauca paleoriver, would have been less affected by the volcanic events associated with the collapse of the Parinacota volcano and the population of *Orestias* have remained *in situ* in the wetlands that were separated from the river [37], safeguarding its genetic diversity. Based on our results, the Parinacota locality may thus be considered as a refuge of the population of the Lauca paleoriver when the catastrophic events affected the LNP zone at the beginning of the Holocene. Although the theory of refuges has been developed mainly in relation to the glacial cycles of the Quaternary [71], the Parinacota population fulfills the characteristics described in this model, such as the detection of greater diversity and the presence of haplotypes shared with peripheral populations that show an expansion signal.

The results based on the microsatellite markers identified Parinacota as a singular population in terms of its genetic diversity. However, in contrast to the mtDNA information that identified Parinacota as a source population for the historical colonization of the other sites, microsatellites markers indicated that Parinacota would correspond currently to a point of admixture of the different genetic groups of the LNP (Fig 5a). In the STRUCTURE analysis, Parinacota population that did not form part of a genetic cluster, instead almost all individuals sampled showed an admixture of the different groups identified (Fig 5a). As the lowest point in the system, the Parinacota wetlands may receive migrants from the other localities in years with inundations, allowing asymmetrical gene flow from the other aquatic systems to the wetlands. This particular connectivity pattern among the different environments suggests that the Parinacota locality is a sink population, receiving the current genetic diversity of the other populations.

The chronology of the vicariance events in the LNP determined that the separation of the populations of *Orestias* was stepwise. First, the volcanic collapse of Parinacota (12500 YBP) [35–38] would have initiated the separation of the populations of the watershed of the Lauca paleoriver in Chungará, Cotacotani and Lauca. Later, at the end of the Pleistocene, the wet episodes would have facilitated the arrival of individuals to the Chungará subwatershed, which is when the separation of *O*. *laucaensis* (Lauca) and *O*. *chungarensis* (Chungará) apparently occurred. Volcanic eruptions processes in the Holocene (8000–4000 YBP) maintained the populations separated, facilitating genetic differentiation between them. During this time there was also a drastic climate change with the arid episode (7000–6000 YBP), in which the connections between the rivers, wetlands and lagoons would have been affected. This is the case of the Piacota lagoon, which was probably separated from the Parinacota wetland by the decrease in water between them and also the eruption of the Ajata volcano, leaving the Piacota lagoon with an isolated population described as *O*. *piacotensis*. Finally, the return of wet periods after 4000 YBP allowed the re-connection of the Cotacotani lagoons with the Lauca river, which is shown by the presence of *O*. *laucaensis* in the different systems as one genetic group.

## Conclusions

The combination of the chronology of the vicariance processes in the LNP and the genetic analyses allows us to conclude that although the localities of the LNP analyzed are not evolutionarily separated units, they are groups in the process of differentiation. The short time since the events that originated this process has not allowed a complete lineage sorting among these populations in spite of the morphological differences that have been described and that motivated the description of different nominal species. In spite of the genetic and phylogeographic differentiation detected in this study, up to now there is no evidence of reproductive isolation among the groups and they do not appear to have developed ecological specialization, at least not at the trophic level [43]. There are also indications that individuals of these groups could maintain contact in the Parinacota wetland by sporadic migrations. It is probable that the different populations of *Orestias* in the LNP are illustrating an uncertain phase of speciation in which sudden changes in the environmental conditions, in this case mainly hydrographic, could reinforce or revert this speciation process, indicating the reticulate nature of evolution [72]. In this context, despite the differences reported in karyotypes in a previous study (Vila et al. 2011), our results support the hypothesis that the morphospecies described in LNP may just correspond to genetically differentiated populations of a same species, together with the overlap of meristic, lack of monophyly and similar dietary habits. Finally, *Orestias* from the LNP appear as an ideal model to exemplify the processes of genetic, morphological, ecological differentiation that accompany an incipient geographic isolation, and thus understand incipient steps of the allopatric speciation process.

## Supporting information

S1 File Google Earth Placemark. Google Earth Placemark file that contains latitudinal and longitudinal coordinates of our sampling sites.
(ZIP)

S2 File Characterístics of the microsatellite loci used for Orestias of the Parque Nacional Lauca. Modified from Esquer et al. 2011. T°a: annealing temperature (°C) (Table A). SAMOVA analysis Spatial molecular analysis of variance (SAMOVA) of D-loop sequences in different geographic groups (Table B). TMRCA. Time to most recent common ancestor using Bayesian skyline analysis for the three genetic groups with expansion signal (Table C). FIS-values for eight microsatellite loci in populations of Orestias of the Lauca National Park (Table D). Genotypes of each sample in each localities of LNP (Table E).
(DOCX)
