# Correction: Pattern of genetic differentiation of an incipient speciation process: The case of the high Andean killifish *Orestias*

**DOI:** 10.1371/journal.pone.0177498

**Published:** 2017-05-08

**Authors:** Claudia Jimena Guerrero-Jiménez, Fabiola Peña, Pamela Morales, Marco A. Méndez, Michel Sallaberry, Irma Vila, Elie Poulin

There are errors in the Funding section. The correct funding information is as follows: Support was provided by Fondecyt Projects 1110243,1140543, 1140540, 1080390 and 1110188. the Instituto de Ecología y Biodiversidad de Chile IEB, ICMP05002 and PFB 023.CONICYT-PCHA/doctorado Nacional/2012-21120972.

The middle initial of the fourth author's name is omitted. The correct name is: Marco A. Méndez. The correct citation is: Guerrero-Jiménez CJ, Peña F, Morales P, Méndez MA, Sallaberry M, Vila I, et al. (2017) Pattern of genetic differentiation of an incipient speciation process: The case of the high Andean killifish *Orestias*. PLoS ONE 12(2): e0170380. https://doi.org/10.1371/journal.pone.0170380
