# Supplementary material for: Pattern of genetic differentiation of an incipient speciation process: The case of the high Andean killifish Orestias
Source: PLoS One. 2017 Feb 28;12(2):e0170380. doi: 10.1371/journal.pone.0170380 (PMC5330459; doi:10.1371/journal.pone.0170380)
Supplement: S2 File — Modified from Esquer et al. 2011. T°a: annealing temperature (°C) (Table A). SAMOVA analysis Spatial molecular analysis of variance (SAMOVA) of D-loop sequences in different geographic groups (Table B). TMRCA. Time to most recent common ancestor using Bayesian skyline analysis for the three genetic groups with expansion signal (Table C). FIS-values for eight microsatellite loci in populations of Orestias of the Lauca National Park (Table D). Genotypes of each sample in each localities of LNP (Table E). (DOCX) [file pone.0170380.s002.docx]

**Supporting Information**

Table A**.** Characterístics of the microsatellite loci used for *Orestias* of the Parque Nacional Lauca. Modified from Esquer et al. 2011. Tºa: annealing temperature (ºC).

| **Locus** | **Repeated Motif** | **Primers(5’–3’)** | **Dye** | **Tºa** |
| --- | --- | --- | --- | --- |
| **A106** | **CA_68_** | **F: TGGCTGATGGTATTGGTTG**  **R: ^a^ AGCACACCTTCACAGGATG** | **VIC** | **58** |
| **A116** | **CA_5_GC_1_CA_6_CC_1_CA_14_** | **F: TCGCTACTTACTCCGACCTC**  **R: AAATCACAATGGCTTTCTCTG** | **PET** | **54** |
| **B1** | **CT_2_TT_1_CT_14_** | **F: TACAAACACATCCATCTCAGTC**  **R: AACACTCCTATCATCCATCATC** | **PET** | **58** |
| **B103** | **CT_3_CCATCG_1_CT_14_** | **F: TATTATCCACTCCTGGTCAGTC**  **R: GTTGAAGCGTTTCCAAGAT** | **FAM** | **51** |
| **B104** | **CT_2_CG_1_CT_4…_CT_10_** | **F: ACCGTAGTTGCCTGGTTACA**  **R: AGGGTGCTGTCAGAGATGAG** | **VIC** | **63** |
| **C102** | **CCAT_15_** | **F: TTCCAAACCACATTTTAGATCC**  **R: CAGCCTTTTGATTATGGAGGT** | **NED** | **62** |
| **C105** | **CCAT_1_CCAA_1_CCAT_8_** | **F: AGCAAGACCAGTTTGAAATCT**  **R: GTTGCCCTGCGATGTAC** | **PET** | **58** |
| **D110** | **GATA_26_** | **F: ATCACAAGACGAGGTTCTCAC**  **R: GATTGGAGCAAGGGACTG** | **FAM** | **58** |
| **A9a** | **CA_5_AA_1_CA_11_** | **F: CAGGAAGGAATCTCAGGAATG**  **R: ACGCACCGTTTCATAGTAAGG** | **FAM** | **58** |
| **C101** | **CCAT_25_CCAC_2_CCAT_2_** | **F: ATTTCAGCAGAGTTTTTGGTAC**  **R:AGCTTCTACTCAGACAAGAGGA** | **NED** | **57** |

Table B. Spatial molecular analysis of variance (SAMOVA) of D-loop sequences in different geographic groups. 1=Chungará, 2=Parinacota, 3=Piacota, 4=Cotacotani, 5=Lauca, 6= Misituni, 7= Copapujo,8= Chuviri. *P< 0.05; **P<0.01; *** P<0.0001.

| **Groups (Localities)** | **% Total variance among groups** | **% Variance among  populations within groups** |
| --- | --- | --- |
| **2 groups**  **3,(1+2+4+5+6+7+8)** | **51.39** | **17.68***** |
| **3 groups**  **1,3,(2+4+5+6+7+8)** | **54.49*** | **10.64***** |
| **4 groups:**  **1,2,3,(4+5+6+7+8)** | **59.92**** | **2.14***** |
| **5 groups**  **1,2,3,(4+5+6+7),8** | **57.65***** | **1.25***** |

Table C. Time to most recent common ancestor using Bayesian skyline analysis for the three genetic groups with expansion signal. Lauca: Lauca+Misituni+Chuviri+Copapujo.

| Genetic Group | TMRCA(years) mean | 95%HPD lower | 95%HPD upper |
| --- | --- | --- | --- |
| Chungará | 21029 | 16892 | 25332 |
| Lauca | 12658 | 10136, | 15224 |
| Parinacota | 39796 | 31781 | 47554 |
| Piacota | 3243 | 2580 | 3874 |

Table D. FIS-values for eight microsatellite loci in populations of *Orestias* of the Lauca National Park. NS= not significant. **= p<0.05. Chung: Chungará; CCC: Chuviri.-Copapujo; Cotacotani; Lau: Lauca-Misituni; Pari: Parinacota; Pia: Piacota.

|  | **Loci** | | | | | | | |
| --- | --- | --- | --- | --- | --- | --- | --- | --- |
| **Population** | **C105** | **A116** | **B1** | **B104** | **D110** | **C102** | **B103** | **A16** |
| **CHUNG** | **-0.04^NS^** | **-0.12 ^NS^** | **-0.22^**^** | **0.01^NS^** | **-0.11^**^** | **1.00^NS^** | **0.13^NS^** | **0.06^NS^** |
| **CCC** | **0.29^**^** | **-0.05 ^NS^** | **0.16^NS^** | **0.12^NS^** | **0.22^NS^** | **0.07^NS^** | **-0.11^NS^** | **-0.04^NS^** |
| **LAU** | **0.21^NS^** | **0.13 ^NS^** | **0.02^NS^** | **0.24^NS^** | **0.03^NS^** | **-0.05^NS^** | **-0.02^NS^** | **-0.05^NS^** |
| **PARI** | **0.05^**^** | **0.21 ^NS^** | **0.08^NS^** | **-0.13^NS^** | **0.16^NS^** | **0.46^NS^** | **0.14^NS^** | **0.00^NS^** |
| **PIA** | **0.04^NS^** | **-0.04 ^NS^** | **-0.05^NS^** | **-0.01^NS^** | **-0.22^NS^** | **0.94^NS^** | **0.33^NS^** | **-0.27^**^** |

Table E. Genotypes of each sample in each localities of LNP.

| Sample | Locus | | | | | | | | | | | | | | | |
| --- | --- | --- | --- | --- | --- | --- | --- | --- | --- | --- | --- | --- | --- | --- | --- | --- |
|  | C105 | | A116 | | B1 | | B104 | | D110 | | C102 | | b103 | | A106 | |
| chungara101 | 220 | 246 | 314 | 316 | 177 | 179 | 145 | 157 | 244 | 248 | 229 | 229 | 195 | 195 | 304 | 306 |
| chungara103 | 208 | 220 | 314 | 316 | 173 | 175 | 145 | 157 | 280 | 292 | 229 | 229 | 193 | 195 | 300 | 300 |
| chungara104 | 220 | 220 | 306 | 312 | 175 | 179 | 145 | 157 | 296 | 300 | 229 | 229 | 193 | 195 | 302 | 306 |
| chungara108 | 220 | 220 | 308 | 318 | 177 | 179 | 145 | 157 | 300 | 332 | 229 | 229 | 193 | 193 | 310 | 312 |
| chungara109 | 220 | 220 | 306 | 316 | 177 | 179 | 145 | 157 | 252 | 292 | 229 | 229 | 193 | 193 | 300 | 304 |
| chungara111 | 220 | 220 | 314 | 314 | 175 | 175 | 145 | 157 | 244 | 248 | 229 | 229 | 193 | 193 | 300 | 304 |
| chungara114 | 208 | 230 | 306 | 316 | 173 | 175 | 145 | 157 | 304 | 328 | 229 | 229 | 193 | 195 | 306 | 308 |
| chungara115 | 212 | 220 | 314 | 320 | 175 | 179 | 157 | 157 | 248 | 252 | 229 | 229 | 193 | 195 | 304 | 304 |
| chungara116 | 208 | 220 | 316 | 316 | 175 | 179 | 157 | 157 | 252 | 292 | 229 | 229 | 193 | 193 | 302 | 304 |
| chungara117 | 220 | 220 | 306 | 316 | 175 | 179 | 157 | 157 | 280 | 296 | 229 | 229 | 193 | 193 | 300 | 300 |
| chungara118 | 208 | 220 | 314 | 316 | 175 | 179 | 157 | 157 | 284 | 292 | 229 | 229 | 193 | 193 | 298 | 300 |
| chungara120 | 208 | 220 | 306 | 314 | 179 | 179 | 157 | 157 | 288 | 300 | 229 | 229 | 193 | 195 | 298 | 302 |
| chungara121 | 220 | 220 | 314 | 316 | 000 | 000 | 000 | 000 | 252 | 296 | 000 | 000 | 193 | 195 | 000 | 000 |
| chungara122 | 208 | 208 | 316 | 318 | 000 | 000 | 157 | 157 | 252 | 296 | 000 | 000 | 193 | 195 | 000 | 000 |
| chungara123 | 208 | 220 | 306 | 314 | 175 | 179 | 157 | 157 | 252 | 284 | 233 | 233 | 193 | 195 | 300 | 304 |
| chungara124 | 208 | 220 | 314 | 316 | 173 | 179 | 155 | 155 | 304 | 320 | 229 | 229 | 193 | 193 | 300 | 304 |
| chungara125 | 208 | 220 | 306 | 316 | 171 | 173 | 157 | 157 | 248 | 292 | 229 | 229 | 193 | 193 | 286 | 300 |
| chungara126 | 208 | 220 | 314 | 316 | 171 | 175 | 157 | 161 | 248 | 284 | 229 | 229 | 193 | 195 | 302 | 304 |
| chungara127 | 208 | 208 | 314 | 316 | 000 | 000 | 145 | 157 | 280 | 292 | 229 | 229 | 195 | 195 | 304 | 308 |
| chungara128 | 220 | 224 | 314 | 316 | 173 | 175 | 157 | 157 | 292 | 296 | 229 | 229 | 193 | 195 | 298 | 302 |
| chungara130 | 208 | 220 | 312 | 322 | 173 | 175 | 145 | 157 | 296 | 300 | 229 | 229 | 195 | 195 | 304 | 304 |
| chungara131 | 220 | 220 | 306 | 320 | 175 | 179 | 145 | 157 | 248 | 292 | 229 | 229 | 193 | 193 | 302 | 306 |
| chungara133 | 216 | 220 | 314 | 314 | 173 | 175 | 145 | 157 | 248 | 292 | 229 | 229 | 193 | 193 | 304 | 308 |
| chungara134 | 220 | 220 | 308 | 318 | 179 | 181 | 159 | 159 | 296 | 300 | 229 | 229 | 195 | 195 | 300 | 300 |
| chuvi101 | 000 | 000 | 314 | 318 | 173 | 173 | 157 | 157 | 252 | 252 | 229 | 233 | 193 | 193 | 294 | 296 |
| chuvi102 | 000 | 000 | 314 | 316 | 173 | 173 | 157 | 157 | 252 | 252 | 229 | 233 | 193 | 193 | 298 | 302 |
| chuvi103 | 208 | 224 | 312 | 314 | 179 | 179 | 157 | 157 | 252 | 252 | 000 | 000 | 000 | 000 | 296 | 306 |
| chuvi104 | 230 | 230 | 314 | 320 | 173 | 173 | 157 | 157 | 252 | 252 | 000 | 000 | 000 | 000 | 298 | 304 |
| chuvi105 | 224 | 230 | 316 | 316 | 173 | 179 | 157 | 157 | 252 | 252 | 229 | 229 | 193 | 193 | 296 | 306 |
| chuvi106 | 220 | 224 | 314 | 316 | 173 | 179 | 157 | 157 | 252 | 252 | 229 | 229 | 193 | 193 | 298 | 302 |
| chuvi107 | 224 | 230 | 314 | 360 | 173 | 179 | 157 | 157 | 252 | 252 | 229 | 229 | 193 | 193 | 296 | 298 |
| chuvi108 | 220 | 224 | 314 | 316 | 177 | 177 | 157 | 157 | 252 | 252 | 229 | 229 | 193 | 193 | 000 | 000 |
| chuvi109 | 224 | 224 | 314 | 356 | 173 | 179 | 157 | 157 | 248 | 252 | 229 | 229 | 193 | 193 | 296 | 306 |
| chuvi110 | 230 | 230 | 314 | 342 | 175 | 179 | 157 | 157 | 252 | 252 | 229 | 233 | 193 | 195 | 298 | 302 |
| chuvi111 | 000 | 000 | 314 | 314 | 175 | 175 | 157 | 157 | 248 | 252 | 229 | 229 | 193 | 211 | 000 | 000 |
| chuvi112 | 000 | 000 | 312 | 316 | 173 | 173 | 157 | 157 | 252 | 252 | 229 | 233 | 193 | 193 | 000 | 000 |
| chuvi113 | 220 | 230 | 314 | 316 | 173 | 175 | 157 | 157 | 284 | 316 | 229 | 229 | 193 | 193 | 296 | 300 |
| chuvi114 | 220 | 234 | 316 | 316 | 173 | 173 | 157 | 157 | 248 | 252 | 229 | 229 | 193 | 193 | 000 | 000 |
| chuvi115 | 216 | 216 | 000 | 000 | 000 | 000 | 000 | 000 | 000 | 000 | 000 | 000 | 193 | 193 | 000 | 000 |
| copa102 | 224 | 224 | 314 | 316 | 173 | 175 | 157 | 157 | 252 | 252 | 000 | 000 | 000 | 000 | 296 | 314 |
| copa103 | 224 | 230 | 312 | 320 | 173 | 175 | 157 | 157 | 248 | 252 | 229 | 229 | 193 | 193 | 298 | 312 |
| copa104 | 000 | 000 | 314 | 344 | 173 | 173 | 157 | 157 | 252 | 256 | 000 | 000 | 000 | 000 | 000 | 000 |
| copa105 | 208 | 224 | 314 | 316 | 173 | 179 | 157 | 157 | 292 | 296 | 229 | 233 | 000 | 000 | 298 | 300 |
| copa106 | 000 | 000 | 316 | 344 | 173 | 179 | 157 | 157 | 252 | 260 | 229 | 229 | 193 | 193 | 000 | 000 |
| copa107 | 224 | 224 | 318 | 342 | 173 | 173 | 157 | 157 | 252 | 252 | 229 | 229 | 193 | 193 | 000 | 000 |
| copa108 | 224 | 224 | 314 | 316 | 173 | 179 | 157 | 157 | 252 | 252 | 229 | 233 | 193 | 195 | 000 | 000 |
| copa109 | 000 | 000 | 312 | 312 | 173 | 179 | 157 | 157 | 252 | 252 | 229 | 229 | 193 | 193 | 000 | 000 |
| copa110 | 000 | 000 | 312 | 314 | 179 | 179 | 157 | 157 | 252 | 260 | 229 | 229 | 193 | 193 | 000 | 000 |
| copa111 | 000 | 000 | 312 | 360 | 173 | 175 | 157 | 157 | 252 | 252 | 229 | 229 | 193 | 195 | 000 | 000 |
| copa112 | 000 | 000 | 314 | 342 | 000 | 000 | 157 | 157 | 252 | 260 | 000 | 000 | 000 | 000 | 000 | 000 |
| copa113 | 000 | 000 | 342 | 344 | 173 | 173 | 157 | 157 | 252 | 252 | 229 | 229 | 193 | 195 | 294 | 296 |
| copa114 | 000 | 000 | 314 | 318 | 173 | 179 | 157 | 157 | 252 | 252 | 229 | 229 | 193 | 195 | 000 | 000 |
| copa115 | 000 | 000 | 342 | 344 | 173 | 173 | 157 | 157 | 252 | 260 | 229 | 229 | 193 | 193 | 296 | 300 |
| copa116 | 220 | 230 | 314 | 316 | 173 | 175 | 157 | 157 | 252 | 252 | 229 | 229 | 193 | 193 | 000 | 000 |
| copa117 | 224 | 224 | 314 | 318 | 175 | 179 | 157 | 157 | 248 | 252 | 229 | 229 | 193 | 193 | 000 | 000 |
| copa118 | 000 | 000 | 314 | 342 | 173 | 175 | 157 | 157 | 252 | 252 | 229 | 233 | 193 | 195 | 296 | 296 |
| copa119 | 220 | 220 | 316 | 344 | 000 | 000 | 157 | 157 | 252 | 252 | 229 | 229 | 193 | 193 | 278 | 296 |
| copa120 | 000 | 000 | 314 | 342 | 179 | 179 | 157 | 157 | 248 | 252 | 229 | 233 | 193 | 195 | 278 | 304 |
| cot101 | 212 | 224 | 314 | 360 | 000 | 000 | 157 | 157 | 252 | 324 | 229 | 229 | 000 | 000 | 296 | 304 |
| cotaco102 | 220 | 230 | 314 | 314 | 173 | 173 | 157 | 157 | 252 | 324 | 229 | 229 | 193 | 195 | 306 | 310 |
| cotaco103 | 230 | 230 | 312 | 318 | 173 | 173 | 157 | 157 | 304 | 312 | 000 | 000 | 000 | 000 | 296 | 306 |
| cotaco104 | 224 | 224 | 314 | 358 | 000 | 000 | 157 | 157 | 284 | 300 | 229 | 233 | 193 | 195 | 296 | 304 |
| cotaco105 | 224 | 230 | 312 | 356 | 173 | 173 | 157 | 157 | 276 | 320 | 229 | 229 | 193 | 193 | 292 | 306 |
| cotaco106 | 193 | 224 | 314 | 320 | 000 | 000 | 155 | 159 | 252 | 304 | 000 | 000 | 000 | 000 | 296 | 300 |
| cotaco107 | 230 | 230 | 314 | 314 | 000 | 000 | 157 | 157 | 300 | 320 | 229 | 233 | 193 | 197 | 300 | 302 |
| cotaco108 | 220 | 230 | 352 | 354 | 175 | 175 | 157 | 157 | 300 | 308 | 229 | 229 | 193 | 193 | 296 | 298 |
| cotaco109 | 224 | 224 | 314 | 316 | 000 | 000 | 157 | 157 | 288 | 288 | 229 | 237 | 193 | 195 | 290 | 306 |
| cotaco110 | 220 | 220 | 354 | 356 | 173 | 173 | 157 | 157 | 304 | 312 | 229 | 229 | 193 | 197 | 296 | 304 |
| cotaco111 | 220 | 220 | 312 | 318 | 173 | 173 | 157 | 157 | 304 | 312 | 000 | 000 | 193 | 193 | 300 | 302 |
| cotaco112 | 224 | 224 | 314 | 356 | 173 | 173 | 157 | 157 | 284 | 300 | 000 | 000 | 193 | 193 | 296 | 304 |
| cotaco113 | 000 | 000 | 310 | 314 | 173 | 173 | 157 | 157 | 252 | 252 | 000 | 000 | 193 | 193 | 306 | 306 |
| cotaco114 | 000 | 000 | 314 | 358 | 173 | 175 | 157 | 157 | 252 | 252 | 229 | 229 | 193 | 193 | 296 | 298 |
| cotaco115 | 000 | 000 | 322 | 352 | 175 | 175 | 157 | 157 | 252 | 252 | 000 | 000 | 193 | 195 | 000 | 000 |
| cotaco116 | 000 | 000 | 314 | 314 | 173 | 173 | 155 | 157 | 252 | 252 | 229 | 233 | 193 | 193 | 000 | 000 |
| cotaco117 | 000 | 000 | 312 | 314 | 173 | 173 | 155 | 157 | 252 | 252 | 229 | 233 | 193 | 193 | 000 | 000 |
| cotaco119 | 000 | 000 | 314 | 318 | 173 | 173 | 157 | 157 | 000 | 000 | 229 | 229 | 193 | 195 | 310 | 314 |
| cotaco120 | 224 | 224 | 312 | 356 | 173 | 173 | 157 | 157 | 252 | 252 | 229 | 229 | 193 | 193 | 298 | 300 |
| cotaco121 | 000 | 000 | 314 | 356 | 173 | 173 | 157 | 157 | 252 | 252 | 233 | 233 | 193 | 193 | 300 | 302 |
| cotaco122 | 000 | 000 | 312 | 312 | 173 | 173 | 157 | 157 | 252 | 252 | 000 | 000 | 193 | 193 | 000 | 000 |
| cotaco123 | 000 | 000 | 318 | 360 | 173 | 175 | 157 | 157 | 252 | 252 | 229 | 229 | 193 | 193 | 300 | 302 |
| cotaco124 | 000 | 000 | 312 | 314 | 173 | 173 | 157 | 157 | 252 | 252 | 229 | 233 | 193 | 193 | 294 | 294 |
| cotaco125 | 000 | 000 | 000 | 000 | 173 | 173 | 157 | 157 | 252 | 252 | 229 | 229 | 193 | 193 | 294 | 296 |
| cotaco126 | 000 | 000 | 312 | 356 | 173 | 175 | 157 | 157 | 252 | 252 | 229 | 229 | 185 | 195 | 300 | 300 |
| cotaco127 | 000 | 000 | 000 | 000 | 173 | 175 | 157 | 157 | 252 | 252 | 229 | 229 | 193 | 193 | 284 | 302 |
| cotaco128 | 000 | 000 | 314 | 352 | 173 | 175 | 157 | 157 | 252 | 252 | 233 | 233 | 193 | 193 | 292 | 316 |
| cotaco130 | 000 | 000 | 314 | 360 | 173 | 175 | 157 | 157 | 252 | 252 | 233 | 237 | 193 | 195 | 294 | 298 |
| cotaco132 | 000 | 000 | 314 | 318 | 173 | 173 | 157 | 157 | 252 | 252 | 229 | 233 | 193 | 195 | 296 | 296 |
| cotaco133 | 224 | 224 | 314 | 314 | 173 | 173 | 157 | 159 | 252 | 252 | 229 | 229 | 193 | 193 | 300 | 306 |
| cotaco134 | 224 | 224 | 356 | 364 | 173 | 173 | 157 | 157 | 252 | 252 | 229 | 229 | 193 | 193 | 298 | 300 |
| cotaco135 | 220 | 224 | 314 | 314 | 173 | 175 | 157 | 157 | 252 | 252 | 000 | 000 | 193 | 195 | 298 | 302 |
| cotaco136 | 000 | 000 | 312 | 364 | 173 | 173 | 157 | 157 | 248 | 252 | 229 | 229 | 193 | 195 | 296 | 304 |
| cotaco137 | 000 | 000 | 000 | 000 | 173 | 175 | 157 | 157 | 248 | 252 | 229 | 229 | 193 | 193 | 296 | 300 |
| cotaco138 | 000 | 000 | 314 | 318 | 173 | 175 | 157 | 157 | 000 | 000 | 229 | 233 | 193 | 195 | 298 | 302 |
| cotaco139 | 212 | 224 | 312 | 320 | 173 | 173 | 157 | 157 | 252 | 252 | 229 | 229 | 193 | 193 | 288 | 302 |
| cotaco140 | 220 | 224 | 312 | 314 | 173 | 175 | 157 | 157 | 252 | 252 | 229 | 233 | 193 | 193 | 296 | 302 |
| cotaco201 | 224 | 224 | 314 | 316 | 173 | 175 | 157 | 157 | 000 | 000 | 233 | 237 | 193 | 193 | 298 | 306 |
| cotaco202 | 224 | 230 | 314 | 318 | 000 | 000 | 157 | 157 | 272 | 292 | 229 | 229 | 193 | 193 | 296 | 308 |
| cotaco203 | 220 | 224 | 316 | 362 | 000 | 000 | 157 | 163 | 300 | 300 | 229 | 229 | 193 | 193 | 300 | 308 |
| cotaco204 | 224 | 224 | 314 | 316 | 000 | 000 | 157 | 157 | 300 | 312 | 229 | 229 | 185 | 193 | 298 | 304 |
| cotaco205 | 193 | 224 | 000 | 000 | 173 | 173 | 157 | 159 | 256 | 316 | 229 | 229 | 193 | 195 | 300 | 302 |
| cotaco206 | 224 | 224 | 312 | 314 | 173 | 173 | 157 | 157 | 252 | 304 | 229 | 233 | 193 | 193 | 298 | 300 |
| cotaco207 | 224 | 224 | 314 | 314 | 000 | 000 | 157 | 157 | 272 | 292 | 229 | 229 | 193 | 193 | 300 | 302 |
| cotaco208 | 224 | 224 | 312 | 314 | 000 | 000 | 157 | 157 | 252 | 304 | 229 | 229 | 193 | 193 | 300 | 314 |
| laus101 | 193 | 230 | 000 | 000 | 173 | 179 | 157 | 157 | 252 | 272 | 000 | 000 | 193 | 193 | 310 | 310 |
| laus102 | 224 | 224 | 000 | 000 | 000 | 000 | 000 | 000 | 000 | 000 | 229 | 229 | 193 | 193 | 296 | 298 |
| laus103 | 220 | 230 | 000 | 000 | 173 | 179 | 157 | 157 | 248 | 296 | 229 | 233 | 193 | 193 | 296 | 306 |
| laus104 | 220 | 220 | 314 | 316 | 000 | 000 | 157 | 157 | 300 | 308 | 229 | 229 | 193 | 193 | 296 | 298 |
| laus105 | 220 | 220 | 316 | 318 | 173 | 173 | 157 | 157 | 296 | 308 | 229 | 229 | 193 | 193 | 000 | 000 |
| laus106 | 220 | 220 | 316 | 316 | 000 | 000 | 157 | 157 | 300 | 316 | 000 | 000 | 193 | 193 | 000 | 000 |
| laus107 | 224 | 230 | 316 | 318 | 000 | 000 | 157 | 157 | 280 | 308 | 229 | 229 | 193 | 193 | 000 | 000 |
| laus108 | 208 | 208 | 316 | 318 | 000 | 000 | 157 | 157 | 292 | 296 | 229 | 229 | 193 | 193 | 000 | 000 |
| laus109 | 220 | 230 | 316 | 316 | 173 | 175 | 157 | 157 | 304 | 316 | 229 | 229 | 193 | 193 | 296 | 298 |
| laus110 | 193 | 230 | 318 | 360 | 171 | 175 | 157 | 157 | 280 | 296 | 229 | 229 | 193 | 193 | 296 | 300 |
| laus111 | 220 | 224 | 316 | 316 | 175 | 179 | 157 | 157 | 296 | 312 | 000 | 000 | 193 | 193 | 300 | 310 |
| laus112 | 208 | 220 | 000 | 000 | 173 | 175 | 157 | 157 | 280 | 316 | 229 | 233 | 193 | 193 | 296 | 310 |
| laus113 | 220 | 234 | 314 | 314 | 175 | 179 | 157 | 157 | 296 | 308 | 229 | 229 | 193 | 193 | 296 | 300 |
| laus114 | 234 | 234 | 314 | 314 | 000 | 000 | 157 | 157 | 308 | 312 | 229 | 229 | 193 | 193 | 300 | 310 |
| laus115 | 220 | 230 | 000 | 000 | 000 | 000 | 157 | 157 | 248 | 296 | 229 | 233 | 000 | 000 | 300 | 314 |
| laus116 | 220 | 234 | 316 | 360 | 000 | 000 | 157 | 157 | 000 | 000 | 229 | 233 | 193 | 193 | 296 | 310 |
| laus118 | 234 | 234 | 314 | 316 | 173 | 175 | 157 | 157 | 292 | 296 | 229 | 229 | 000 | 000 | 296 | 298 |
| laus119 | 208 | 220 | 316 | 356 | 175 | 179 | 157 | 157 | 000 | 000 | 229 | 233 | 193 | 203 | 298 | 300 |
| laus120 | 208 | 208 | 316 | 318 | 175 | 179 | 157 | 157 | 308 | 312 | 229 | 229 | 000 | 000 | 296 | 306 |
| laus121 | 208 | 230 | 314 | 342 | 175 | 179 | 157 | 157 | 296 | 300 | 229 | 229 | 193 | 193 | 000 | 000 |
| laus122 | 230 | 230 | 318 | 362 | 175 | 179 | 157 | 157 | 248 | 248 | 229 | 229 | 193 | 193 | 296 | 314 |
| laus123 | 208 | 230 | 000 | 000 | 173 | 179 | 157 | 157 | 248 | 304 | 229 | 229 | 193 | 193 | 308 | 310 |
| laus124 | 220 | 234 | 316 | 362 | 173 | 179 | 157 | 157 | 288 | 308 | 229 | 229 | 000 | 000 | 296 | 300 |
| laus125 | 224 | 230 | 316 | 316 | 000 | 000 | 157 | 157 | 248 | 316 | 229 | 229 | 193 | 193 | 296 | 310 |
| laus126 | 208 | 208 | 316 | 356 | 173 | 175 | 157 | 157 | 296 | 304 | 229 | 229 | 000 | 000 | 300 | 300 |
| laus127 | 208 | 230 | 342 | 356 | 173 | 175 | 157 | 157 | 288 | 288 | 229 | 229 | 193 | 193 | 308 | 310 |
| laus128 | 208 | 208 | 316 | 316 | 173 | 175 | 157 | 157 | 312 | 316 | 229 | 233 | 193 | 193 | 300 | 306 |
| laus129 | 220 | 230 | 314 | 314 | 173 | 175 | 157 | 157 | 284 | 288 | 229 | 233 | 000 | 000 | 296 | 298 |
| laus130 | 220 | 230 | 314 | 314 | 175 | 179 | 157 | 157 | 296 | 308 | 229 | 229 | 193 | 193 | 298 | 300 |
| laus131 | 208 | 220 | 314 | 314 | 173 | 173 | 157 | 157 | 304 | 308 | 229 | 229 | 193 | 193 | 308 | 310 |
| laus132 | 208 | 220 | 316 | 316 | 173 | 179 | 157 | 157 | 300 | 312 | 229 | 229 | 193 | 193 | 296 | 310 |
| laus134 | 220 | 220 | 314 | 314 | 173 | 173 | 157 | 157 | 000 | 000 | 229 | 229 | 193 | 193 | 298 | 306 |
| laus135 | 230 | 230 | 316 | 316 | 173 | 179 | 157 | 157 | 296 | 304 | 229 | 229 | 193 | 193 | 296 | 310 |
| laus136 | 220 | 230 | 356 | 362 | 173 | 175 | 157 | 157 | 248 | 308 | 229 | 233 | 193 | 193 | 296 | 296 |
| laus201 | 208 | 224 | 314 | 316 | 175 | 179 | 157 | 157 | 248 | 288 | 229 | 229 | 193 | 193 | 308 | 310 |
| laus202 | 208 | 220 | 314 | 362 | 173 | 179 | 157 | 157 | 308 | 308 | 229 | 233 | 193 | 193 | 298 | 300 |
| laus203 | 208 | 234 | 316 | 316 | 179 | 179 | 157 | 157 | 248 | 308 | 229 | 233 | 193 | 193 | 298 | 300 |
| laus204 | 220 | 220 | 314 | 316 | 173 | 173 | 157 | 157 | 296 | 308 | 229 | 229 | 193 | 193 | 294 | 296 |
| laus205 | 224 | 230 | 314 | 356 | 173 | 173 | 157 | 157 | 248 | 248 | 229 | 233 | 193 | 193 | 296 | 302 |
| laus206 | 224 | 224 | 314 | 314 | 173 | 175 | 159 | 163 | 244 | 300 | 229 | 229 | 193 | 193 | 294 | 296 |
| laus208 | 204 | 230 | 316 | 318 | 175 | 179 | 157 | 157 | 308 | 320 | 000 | 000 | 193 | 193 | 000 | 000 |
| laus209 | 208 | 230 | 312 | 314 | 173 | 179 | 157 | 157 | 280 | 300 | 229 | 229 | 193 | 193 | 298 | 300 |
| laus210 | 220 | 230 | 312 | 314 | 173 | 175 | 157 | 157 | 288 | 300 | 229 | 229 | 193 | 195 | 300 | 304 |
| laus211 | 224 | 230 | 312 | 312 | 175 | 175 | 157 | 161 | 248 | 248 | 229 | 233 | 193 | 193 | 296 | 298 |
| laus212 | 230 | 230 | 360 | 362 | 173 | 179 | 157 | 157 | 308 | 316 | 229 | 229 | 193 | 193 | 296 | 296 |
| laus213 | 220 | 224 | 312 | 316 | 173 | 173 | 157 | 157 | 000 | 000 | 233 | 233 | 193 | 197 | 298 | 298 |
| laus214 | 230 | 234 | 316 | 362 | 175 | 175 | 157 | 157 | 308 | 312 | 229 | 229 | 193 | 193 | 300 | 312 |
| laus215 | 234 | 234 | 314 | 314 | 173 | 173 | 157 | 157 | 300 | 300 | 229 | 233 | 193 | 193 | 000 | 000 |
| laus216 | 206 | 224 | 316 | 318 | 000 | 000 | 157 | 159 | 300 | 308 | 229 | 233 | 193 | 193 | 300 | 300 |
| laus217 | 230 | 230 | 316 | 316 | 179 | 179 | 157 | 157 | 292 | 312 | 229 | 229 | 193 | 193 | 296 | 300 |
| laus218 | 224 | 230 | 314 | 362 | 000 | 000 | 157 | 157 | 300 | 300 | 229 | 233 | 193 | 193 | 296 | 308 |
| laus220 | 220 | 224 | 304 | 314 | 173 | 173 | 157 | 157 | 288 | 292 | 229 | 229 | 193 | 193 | 296 | 304 |
| laus221 | 220 | 220 | 314 | 356 | 173 | 179 | 157 | 157 | 244 | 248 | 229 | 229 | 193 | 193 | 296 | 310 |
| laus222 | 208 | 220 | 314 | 316 | 173 | 175 | 157 | 157 | 296 | 308 | 229 | 229 | 193 | 193 | 298 | 300 |
| laus223 | 230 | 230 | 314 | 362 | 175 | 179 | 157 | 157 | 296 | 312 | 229 | 233 | 193 | 193 | 296 | 300 |
| laus224 | 208 | 208 | 314 | 314 | 173 | 173 | 157 | 157 | 248 | 308 | 229 | 233 | 193 | 195 | 298 | 300 |
| laus234 | 220 | 230 | 316 | 318 | 179 | 179 | 157 | 157 | 296 | 308 | 229 | 229 | 193 | 193 | 296 | 296 |
| laus236 | 230 | 234 | 314 | 356 | 175 | 179 | 157 | 157 | 296 | 312 | 229 | 229 | 193 | 193 | 000 | 000 |
| laus237 | 208 | 208 | 312 | 314 | 173 | 173 | 157 | 157 | 288 | 308 | 229 | 233 | 193 | 193 | 300 | 302 |
| laus238 | 230 | 230 | 316 | 360 | 175 | 179 | 157 | 157 | 248 | 304 | 229 | 229 | 193 | 193 | 294 | 296 |
| laus239 | 220 | 220 | 314 | 362 | 000 | 000 | 157 | 157 | 304 | 312 | 229 | 233 | 193 | 195 | 294 | 296 |
| laus241 | 193 | 224 | 314 | 356 | 000 | 000 | 157 | 157 | 308 | 308 | 229 | 229 | 193 | 193 | 300 | 304 |
| laus242 | 224 | 224 | 312 | 316 | 000 | 000 | 157 | 157 | 308 | 320 | 229 | 229 | 193 | 193 | 296 | 300 |
| laus246 | 220 | 230 | 316 | 318 | 173 | 175 | 157 | 157 | 000 | 000 | 229 | 229 | 193 | 193 | 000 | 000 |
| laus247 | 208 | 220 | 312 | 316 | 000 | 000 | 157 | 157 | 248 | 312 | 229 | 229 | 193 | 193 | 300 | 302 |
| laus248 | 208 | 230 | 316 | 318 | 173 | 173 | 157 | 157 | 288 | 300 | 229 | 229 | 193 | 193 | 300 | 302 |
| laus249 | 208 | 220 | 314 | 360 | 173 | 173 | 157 | 157 | 288 | 308 | 229 | 229 | 193 | 193 | 300 | 308 |
| laus_225 | 208 | 220 | 316 | 318 | 000 | 000 | 157 | 157 | 000 | 000 | 229 | 229 | 193 | 193 | 296 | 298 |
| laus_226 | 220 | 220 | 312 | 314 | 173 | 175 | 157 | 157 | 284 | 296 | 229 | 229 | 193 | 193 | 292 | 302 |
| laus_227 | 224 | 224 | 312 | 312 | 173 | 173 | 157 | 157 | 000 | 000 | 229 | 229 | 193 | 193 | 298 | 312 |
| laus_228 | 220 | 224 | 000 | 000 | 000 | 000 | 157 | 157 | 308 | 316 | 229 | 229 | 193 | 193 | 300 | 302 |
| laus_229 | 224 | 224 | 314 | 314 | 173 | 173 | 157 | 157 | 304 | 334 | 229 | 229 | 193 | 193 | 300 | 310 |
| laus_230 | 208 | 234 | 316 | 316 | 173 | 173 | 157 | 157 | 300 | 308 | 229 | 229 | 193 | 193 | 296 | 310 |
| laus_231 | 230 | 234 | 314 | 316 | 173 | 173 | 157 | 157 | 304 | 312 | 229 | 229 | 193 | 193 | 296 | 296 |
| laus_232 | 224 | 230 | 000 | 000 | 000 | 000 | 157 | 157 | 308 | 308 | 229 | 229 | 193 | 193 | 296 | 296 |
| laus_244 | 224 | 230 | 314 | 318 | 000 | 000 | 157 | 157 | 000 | 000 | 229 | 229 | 193 | 193 | 302 | 306 |
| pari101 | 230 | 230 | 314 | 314 | 173 | 175 | 145 | 155 | 304 | 316 | 229 | 229 | 193 | 193 | 300 | 310 |
| pari102 | 230 | 230 | 314 | 314 | 175 | 177 | 157 | 159 | 252 | 252 | 229 | 229 | 193 | 193 | 308 | 310 |
| pari103 | 193 | 230 | 316 | 360 | 173 | 179 | 145 | 157 | 304 | 308 | 229 | 233 | 193 | 193 | 310 | 310 |
| pari104 | 000 | 000 | 314 | 314 | 173 | 175 | 157 | 157 | 248 | 252 | 229 | 229 | 185 | 189 | 310 | 312 |
| pari105 | 224 | 230 | 316 | 318 | 175 | 175 | 145 | 157 | 300 | 304 | 233 | 233 | 000 | 000 | 296 | 298 |
| pari107 | 193 | 230 | 314 | 318 | 173 | 177 | 157 | 157 | 308 | 312 | 229 | 229 | 193 | 193 | 310 | 310 |
| pari108 | 230 | 230 | 314 | 316 | 175 | 179 | 145 | 157 | 292 | 300 | 229 | 229 | 193 | 195 | 310 | 312 |
| pari109 | 193 | 230 | 314 | 314 | 173 | 175 | 145 | 157 | 288 | 300 | 229 | 229 | 000 | 000 | 300 | 308 |
| pari111 | 230 | 230 | 316 | 320 | 173 | 179 | 145 | 157 | 296 | 304 | 229 | 229 | 189 | 193 | 300 | 306 |
| pari112 | 224 | 230 | 312 | 314 | 175 | 179 | 145 | 157 | 308 | 324 | 229 | 229 | 193 | 193 | 296 | 310 |
| pari201 | 224 | 230 | 314 | 314 | 173 | 173 | 145 | 157 | 300 | 304 | 229 | 233 | 193 | 193 | 304 | 314 |
| pari202 | 220 | 230 | 308 | 316 | 173 | 173 | 145 | 157 | 292 | 304 | 229 | 229 | 000 | 000 | 300 | 314 |
| pari203 | 224 | 230 | 314 | 314 | 175 | 175 | 157 | 157 | 292 | 308 | 229 | 229 | 000 | 000 | 300 | 302 |
| pari204 | 220 | 230 | 314 | 314 | 173 | 175 | 145 | 157 | 304 | 308 | 229 | 229 | 193 | 193 | 300 | 300 |
| pari205 | 230 | 230 | 320 | 356 | 173 | 173 | 157 | 157 | 296 | 304 | 229 | 229 | 193 | 193 | 300 | 306 |
| pari206 | 230 | 230 | 314 | 314 | 175 | 179 | 157 | 157 | 292 | 300 | 229 | 229 | 000 | 000 | 300 | 314 |
| pari207 | 224 | 230 | 314 | 320 | 173 | 175 | 157 | 159 | 248 | 252 | 229 | 229 | 000 | 000 | 298 | 300 |
| pari208 | 224 | 230 | 316 | 356 | 175 | 179 | 157 | 157 | 252 | 260 | 233 | 233 | 000 | 000 | 300 | 314 |
| pari209 | 230 | 230 | 314 | 314 | 175 | 179 | 145 | 157 | 252 | 252 | 233 | 233 | 000 | 000 | 300 | 300 |
| pari210 | 193 | 230 | 314 | 316 | 173 | 175 | 145 | 157 | 252 | 256 | 229 | 229 | 193 | 193 | 302 | 306 |
| pari211 | 230 | 230 | 312 | 314 | 173 | 173 | 157 | 157 | 252 | 252 | 229 | 229 | 193 | 193 | 300 | 310 |
| pari212 | 230 | 230 | 314 | 320 | 171 | 175 | 157 | 157 | 252 | 252 | 229 | 233 | 193 | 193 | 300 | 314 |
| pari213 | 220 | 230 | 312 | 314 | 173 | 179 | 157 | 157 | 252 | 252 | 229 | 229 | 193 | 193 | 314 | 314 |
| pari214 | 230 | 230 | 318 | 318 | 173 | 173 | 157 | 157 | 244 | 252 | 229 | 229 | 193 | 193 | 300 | 310 |
| pari215 | 000 | 000 | 314 | 316 | 175 | 175 | 157 | 157 | 252 | 252 | 229 | 233 | 193 | 193 | 302 | 308 |
| pari216 | 000 | 000 | 312 | 318 | 173 | 179 | 157 | 157 | 252 | 252 | 229 | 229 | 193 | 193 | 300 | 302 |
| pari217 | 000 | 000 | 314 | 314 | 175 | 179 | 157 | 159 | 252 | 252 | 229 | 229 | 193 | 193 | 300 | 302 |
| pari218 | 224 | 224 | 314 | 314 | 173 | 175 | 157 | 157 | 252 | 252 | 000 | 000 | 193 | 193 | 304 | 308 |
| pari301 | 230 | 230 | 314 | 318 | 175 | 175 | 157 | 157 | 248 | 304 | 229 | 229 | 193 | 195 | 298 | 300 |
| pari302 | 000 | 000 | 314 | 314 | 175 | 175 | 000 | 000 | 000 | 000 | 229 | 229 | 193 | 195 | 300 | 304 |
| pari304 | 000 | 000 | 314 | 316 | 173 | 179 | 157 | 157 | 276 | 300 | 229 | 233 | 193 | 193 | 298 | 300 |
| pari305 | 230 | 230 | 322 | 322 | 173 | 173 | 152 | 159 | 000 | 000 | 229 | 229 | 000 | 000 | 000 | 000 |
| pari306 | 000 | 000 | 322 | 324 | 175 | 175 | 152 | 157 | 240 | 244 | 229 | 229 | 195 | 223 | 296 | 318 |
| pari307 | 212 | 212 | 322 | 322 | 173 | 173 | 152 | 157 | 252 | 252 | 000 | 000 | 193 | 223 | 000 | 000 |
| piacota101 | 230 | 230 | 314 | 316 | 173 | 179 | 157 | 157 | 300 | 304 | 229 | 229 | 193 | 193 | 300 | 310 |
| piacota102 | 208 | 230 | 362 | 364 | 000 | 000 | 157 | 157 | 304 | 312 | 000 | 000 | 193 | 193 | 000 | 000 |
| piacota103 | 000 | 000 | 362 | 366 | 000 | 000 | 000 | 000 | 300 | 304 | 000 | 000 | 193 | 193 | 308 | 310 |
| piacota104 | 230 | 230 | 312 | 316 | 000 | 000 | 157 | 157 | 300 | 304 | 000 | 000 | 193 | 193 | 300 | 310 |
| piacota105 | 230 | 230 | 314 | 320 | 000 | 000 | 157 | 157 | 300 | 304 | 000 | 000 | 193 | 193 | 308 | 310 |
| piacota106 | 000 | 000 | 316 | 320 | 000 | 000 | 000 | 000 | 300 | 304 | 000 | 000 | 193 | 193 | 308 | 310 |
| piacota107 | 208 | 230 | 316 | 320 | 000 | 000 | 157 | 157 | 300 | 312 | 000 | 000 | 193 | 193 | 308 | 310 |
| piacota108 | 208 | 230 | 314 | 320 | 175 | 179 | 157 | 159 | 300 | 304 | 229 | 229 | 193 | 193 | 310 | 310 |
| piacota109 | 230 | 230 | 316 | 320 | 173 | 175 | 157 | 157 | 300 | 300 | 229 | 229 | 193 | 193 | 300 | 310 |
| piacota110 | 230 | 230 | 316 | 320 | 173 | 179 | 157 | 157 | 304 | 312 | 233 | 233 | 193 | 193 | 300 | 310 |
| piacota111 | 230 | 230 | 314 | 316 | 173 | 175 | 145 | 157 | 300 | 312 | 229 | 229 | 193 | 193 | 300 | 310 |
| piacota201 | 230 | 230 | 312 | 314 | 175 | 179 | 000 | 000 | 000 | 000 | 229 | 229 | 193 | 193 | 310 | 310 |
| piacota202 | 208 | 230 | 314 | 320 | 173 | 179 | 157 | 157 | 304 | 304 | 229 | 229 | 193 | 193 | 308 | 310 |
| piacota203 | 000 | 000 | 312 | 320 | 000 | 000 | 000 | 000 | 300 | 304 | 000 | 000 | 193 | 193 | 000 | 000 |
| piacota204 | 208 | 230 | 312 | 312 | 179 | 179 | 157 | 157 | 000 | 000 | 233 | 237 | 195 | 197 | 304 | 304 |
| piacota205 | 230 | 230 | 314 | 320 | 000 | 000 | 157 | 157 | 304 | 312 | 229 | 229 | 193 | 193 | 300 | 310 |
| piacota206 | 000 | 000 | 314 | 366 | 000 | 000 | 000 | 000 | 292 | 304 | 000 | 000 | 193 | 193 | 296 | 310 |
| piacota207 | 230 | 230 | 312 | 318 | 171 | 173 | 157 | 157 | 000 | 000 | 229 | 229 | 193 | 193 | 308 | 310 |
| piacota208 | 230 | 230 | 314 | 364 | 000 | 000 | 000 | 000 | 300 | 304 | 000 | 000 | 193 | 193 | 306 | 310 |
| piacota209 | 230 | 230 | 320 | 364 | 173 | 175 | 157 | 157 | 300 | 304 | 229 | 229 | 193 | 193 | 300 | 310 |
| piacota210 | 000 | 000 | 320 | 366 | 000 | 000 | 000 | 000 | 000 | 000 | 000 | 000 | 193 | 193 | 308 | 310 |
| piacota211 | 230 | 230 | 314 | 316 | 000 | 000 | 000 | 000 | 000 | 000 | 000 | 000 | 193 | 193 | 300 | 310 |
| piacota212 | 230 | 230 | 314 | 366 | 000 | 000 | 000 | 000 | 000 | 000 | 233 | 233 | 193 | 193 | 308 | 310 |
| piacota213 | 230 | 230 | 312 | 320 | 000 | 000 | 000 | 000 | 300 | 304 | 233 | 233 | 193 | 193 | 310 | 314 |
| piacota214 | 208 | 230 | 314 | 364 | 000 | 000 | 157 | 157 | 304 | 308 | 229 | 229 | 193 | 193 | 300 | 310 |
| piacota215 | 230 | 230 | 314 | 314 | 173 | 179 | 157 | 157 | 300 | 304 | 229 | 229 | 193 | 193 | 306 | 308 |
| piacota216 | 230 | 230 | 314 | 316 | 175 | 175 | 157 | 157 | 300 | 312 | 229 | 229 | 193 | 193 | 300 | 310 |
| piacota217 | 000 | 000 | 314 | 316 | 000 | 000 | 000 | 000 | 304 | 304 | 000 | 000 | 193 | 193 | 000 | 000 |
| piacota218 | 208 | 230 | 312 | 314 | 175 | 175 | 157 | 157 | 300 | 304 | 233 | 233 | 193 | 193 | 300 | 308 |
| piacota219 | 230 | 230 | 314 | 314 | 173 | 175 | 157 | 157 | 292 | 300 | 233 | 233 | 189 | 193 | 310 | 312 |
| piacota221 | 208 | 208 | 314 | 316 | 175 | 175 | 157 | 157 | 300 | 300 | 233 | 233 | 193 | 193 | 310 | 310 |
| piacota222 | 000 | 000 | 314 | 320 | 175 | 175 | 157 | 157 | 300 | 312 | 229 | 229 | 193 | 193 | 300 | 310 |
| piacota223 | 224 | 230 | 314 | 314 | 173 | 173 | 157 | 157 | 296 | 304 | 233 | 233 | 193 | 193 | 300 | 310 |
| piacota224 | 230 | 230 | 314 | 364 | 175 | 179 | 157 | 157 | 300 | 312 | 233 | 233 | 193 | 193 | 300 | 310 |
| piacota225 | 230 | 230 | 320 | 320 | 173 | 179 | 157 | 157 | 300 | 304 | 233 | 233 | 193 | 193 | 308 | 310 |
| piacota226 | 224 | 230 | 314 | 316 | 173 | 175 | 157 | 157 | 300 | 304 | 000 | 000 | 193 | 193 | 300 | 300 |
| piacota227 | 208 | 230 | 314 | 314 | 173 | 173 | 157 | 157 | 300 | 300 | 233 | 233 | 193 | 193 | 300 | 310 |
| piacota228 | 208 | 230 | 314 | 316 | 173 | 175 | 157 | 157 | 300 | 304 | 229 | 229 | 193 | 193 | 310 | 310 |
| piacota229 | 208 | 230 | 314 | 314 | 173 | 175 | 157 | 157 | 300 | 304 | 233 | 233 | 193 | 193 | 300 | 310 |
| piacota230 | 208 | 230 | 316 | 320 | 173 | 179 | 157 | 157 | 300 | 304 | 233 | 233 | 193 | 193 | 300 | 310 |
| piacota231 | 230 | 230 | 316 | 320 | 173 | 175 | 157 | 157 | 300 | 300 | 233 | 233 | 193 | 193 | 300 | 310 |
| piacota232 | 208 | 208 | 314 | 320 | 173 | 173 | 157 | 157 | 292 | 304 | 233 | 233 | 193 | 193 | 300 | 310 |
| piacota233 | 230 | 230 | 314 | 320 | 175 | 179 | 157 | 157 | 292 | 304 | 229 | 229 | 193 | 193 | 300 | 310 |
| piacota234 | 208 | 230 | 320 | 320 | 000 | 000 | 157 | 157 | 304 | 304 | 000 | 000 | 193 | 193 | 308 | 310 |
| piacota235 | 230 | 230 | 320 | 320 | 175 | 181 | 157 | 157 | 308 | 312 | 000 | 000 | 193 | 193 | 300 | 300 |
| piacota236 | 230 | 230 | 316 | 364 | 175 | 181 | 157 | 157 | 300 | 308 | 000 | 000 | 193 | 193 | 300 | 310 |
| piacota237 | 000 | 000 | 316 | 364 | 000 | 000 | 000 | 000 | 300 | 308 | 000 | 000 | 193 | 193 | 308 | 310 |
| piacota238 | 230 | 230 | 314 | 316 | 000 | 000 | 000 | 000 | 300 | 304 | 000 | 000 | 193 | 193 | 308 | 310 |
